# Neutrophil-to-HDL cholesterol ratio and cardiovascular disease coexistence in COPD patients: An NHANES 2007 to 2018 analysis

**DOI:** 10.1097/MD.0000000000050771

**Published:** 2026-09-18

**Authors:** Xia Zhang, Juhua Duan

**Affiliations:** a Nanjing Lishui People’s Hospital, Zhongda Hospital Lishui Branch, Southeast University, Nanjing, Jiangsu, China.

**Keywords:** biomarkers, cardiovascular disease, COPD, Inflammation, neutrophil-to-HDL ratio

## Abstract

The neutrophil-to-high-density lipoprotein cholesterol ratio (NHR) is a composite biomarker reflecting systemic inflammation and lipid metabolism. We examined associations between NHR and cardiovascular disease (CVD) in patients with chronic obstructive pulmonary disease (COPD). This cross-sectional study analyzed National Health and Nutrition Examination Survey data (2007–2018) including adults aged ≥40 years with COPD. CVD was defined as self-reported heart failure, coronary artery disease, angina, or myocardial infarction. NHR was calculated as neutrophil count (×10^3^/μL) divided by high-density lipoprotein cholesterol (mmol/L). Multivariable logistic regression assessed NHR–CVD associations, with restricted cubic splines evaluating dose–response relationships. Among 1033 COPD patients, 199 (19.3%) had CVD. After multivariable adjustment, each unit increase in NHR was associated with higher CVD odds ratio (OR: 1.24, 95% confidence interval [CI]: 1.11–1.38). Compared to the lowest quartile, *Q*3 (OR: 1.65, 95% CI: 1.04–2.85) and *Q*4 (OR: 2.60, 95% CI: 1.52–4.52) showed higher CVD odds. Spline analysis revealed a nonlinear relationship with a potential inflection point around NHR ≈ 3.4. Associations remained consistent across subgroups. Elevated NHR is independently associated with higher CVD prevalence in COPD patients. This cross-sectional analysis suggests NHR may serve as an accessible inflammatory biomarker reflecting cardiovascular comorbidity in this population.

## 1. Introduction

Chronic obstructive pulmonary disease (COPD) affects over 250 million people globally and ranks as the 3rd leading cause of death worldwide.^[1]^ Beyond progressive airflow limitation, COPD is now recognized as a systemic inflammatory condition with multiple extrapulmonary manifestations. Cardiovascular disease (CVD) represents the most significant comorbidity, occurring in 20% to 70% of COPD patients and contributing substantially to morbidity and mortality.^[2]^

The COPD–CVD relationship extends beyond shared risk factors such as smoking. Chronic airway inflammation triggers systemic spillover of inflammatory mediators, creating a pro-inflammatory state characterized by elevated acute-phase proteins, cytokines, and coagulation factors.^[3]^ This promotes endothelial dysfunction, accelerates atherosclerosis, and increases thrombotic risk.^[4]^ Additional mechanisms include hypoxia-induced pulmonary hypertension, autonomic dysregulation, and corticosteroid effects.^[5]^

Traditional cardiovascular risk scores (e.g., Framingham, American College of Cardiology/American Heart Association equations) often underperform in COPD patients due to their unique inflammatory profile.^[6]^ This has driven interest in inflammatory biomarkers that better capture COPD-related cardiovascular comorbidity. Hematologic ratios such as neutrophil-to-lymphocyte ratio offer practical advantages – universal availability, standardization, and low cost – while reflecting inflammatory balance.^[7]^

The neutrophil-to-high-density lipoprotein cholesterol (HDL-C) ratio (NHR) combines 2 complementary pathways: innate immune activation, reflected by the neutrophil count, and lipid metabolism, represented by HDL-C. Neutrophils contribute to COPD pathogenesis via proteases, oxidative stress, and extracellular trap formation,^[8]^ while HDL-C exerts anti-inflammatory, antioxidant, and endothelial-protective effects.^[9]^

In COPD, these pathways may interact in ways relevant to cardiovascular comorbidity: neutrophil-mediated mechanisms promote systemic inflammation and atherosclerotic plaque instability,^[10]^ while chronic inflammation may impair HDL function through oxidative modification.^[11]^ NHR therefore captures the balance between pro-inflammatory and anti-inflammatory forces in this population.

NHR has shown prognostic value in acute coronary syndromes and heart failure,^[12]^ yet its relationship with CVD in COPD remains unexplored. Given the heightened inflammatory burden in COPD and the potential for altered HDL function, NHR may be particularly relevant for assessing cardiovascular comorbidity in this population.

We therefore conducted a cross-sectional analysis of National Health and Nutrition Examination Survey (NHANES) data (2007–2018) to examine NHR–CVD associations in COPD patients, hypothesizing that elevated NHR would independently associate with increased CVD prevalence.

## 2. Methods

### 2.1. Study population and design

We performed a cross-sectional analysis utilizing data from the NHANES, a nationally representative health assessment program conducted by the Centers for Disease Control and Prevention. Our analysis incorporated data from 6 consecutive survey cycles spanning 2007 to 2018 (2007–2008, 2009–2010, 2011–2012, 2013–2014, 2015–2016, 2017–2018).

The NHANES program employs a stratified multistage probability sampling framework to obtain nationally representative data from the U.S. civilian noninstitutionalized population.^[13]^ Data collection encompasses standardized questionnaires, clinical examinations, and laboratory assessments conducted in mobile examination centers. The survey protocol received approval from the National Center for Health Statistics Research Ethics Review Board, with written informed consent obtained from all participants.

**Figure 1.**
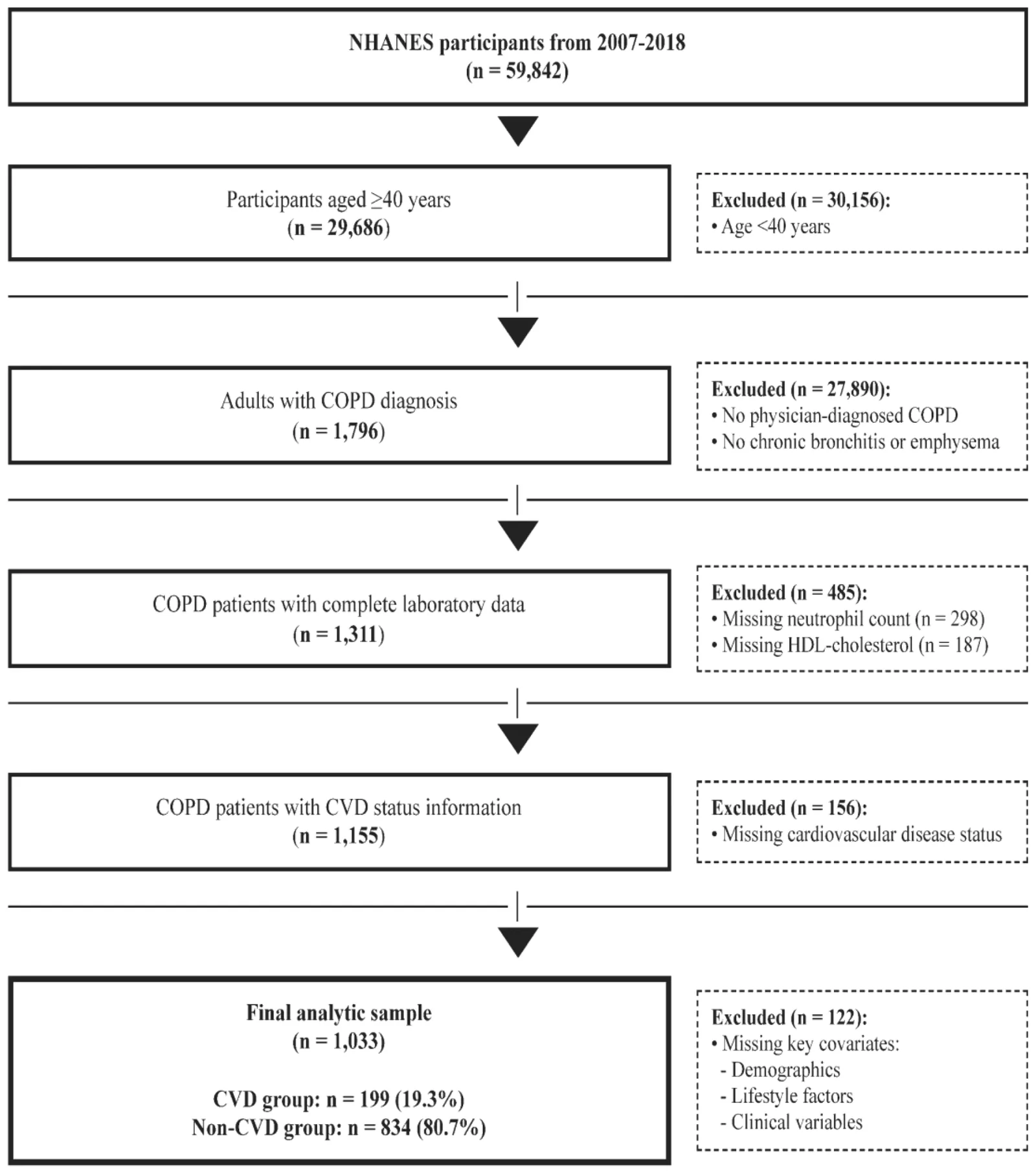
Flow diagram of participant selection process. COPD = chronic obstructive pulmonary disease, CVD = cardiovascular disease, HDL = high-density lipoprotein, NHANES = National Health and Nutrition Examination Survey.

From 59,842 NHANES participants (2007–2018), we included adults aged ≥40 years with a self-reported COPD diagnosis (n = 1796). We excluded participants with missing neutrophil counts (n = 298), missing HDL-C (n = 187), missing cardiovascular disease status (n = 156), or missing key covariates (n = 122). Our final analytical cohort included 1033 adults (Fig. 1). Complete NHANES methodology documentation is available at https://www.cdc.gov/nchs/nhanes.

### 2.2. Variable definitions and measurements

COPD status was ascertained through participant self-report of healthcare provider-diagnosed chronic bronchitis (survey item MCQ160K) and/or emphysema (survey item MCQ160G).^[14]^ This approach aligns with established NHANES-based COPD research and provides good concordance with spirometry-confirmed disease in population studies.

Cardiovascular disease was defined as a self-reported healthcare provider diagnosis of any of the following conditions: heart failure (MCQ160B), coronary artery disease (MCQ160C), angina pectoris (MCQ160D), or acute myocardial infarction (MCQ160E). This composite definition captures the major cardiovascular conditions relevant to COPD patients.^[15]^

The NHR was calculated as absolute neutrophil count (×10^3^ cells/μL) divided by HDL-C concentration (mmol/L). Neutrophil counts were obtained through automated complete blood count analysis using standardized laboratory protocols. HDL-C was measured using direct enzymatic methods following standardized procedures.^[16]^

Demographic variables included age (continuous), sex (male/female), and race/ethnicity (Mexican American, Non-Hispanic Black, Non-Hispanic White, other Hispanic, and other race).^[17]^ Socioeconomic factors comprised education level (categorized as <9th grade, 9th–11th grade, high school graduate, some college or associate degree, and college graduate or above), marital status (married, divorced, living with partner, never married, separated, and widowed), and poverty income ratio (PIR) (continuous, range 0–5).

Clinical variables included smoking status, body mass index (BMI) (continuous, kg/m^2^), and comorbidities. Smoking status was categorized as current smokers (≥100 cigarettes lifetime and currently smoking), former smokers (≥100 cigarettes lifetime but not currently smoking), and never smokers (<100 cigarettes lifetime). For analytical purposes, current and former smokers were combined and compared to never smokers. Hypertension was defined as a self-reported diagnosis or antihypertensive medication use. Diabetes mellitus was defined as a self-reported diagnosis or antidiabetic medication use.^[18,19]^

### 2.3. Statistical analysis

Baseline characteristics were compared between COPD patients with and without cardiovascular disease using chi-square tests for categorical variables and Student *t* tests for continuous variables.

NHR was analyzed both as a continuous variable and categorized into quartiles based on the distribution within our COPD cohort (*Q*1: 0.492–2.32, *Q*2: 2.321–3.361, *Q*3: 3.362–4.677, *Q*4: 4.679–8.624), with the lowest quartile serving as the reference category.

We constructed 4 sequential logistic regression models to assess NHR–CVD associations: model 1 (crude association); model 2 (adjusted for age, sex, and race/ethnicity); model 3 (additionally adjusted for education, marital status, smoking status, and PIR); and model 4 (further adjusted for hypertension, diabetes, and BMI). This modeling approach allows assessment of confounding by different variable sets. We acknowledge that hypertension, diabetes, and obesity may lie partly on the inflammatory pathway linking NHR to CVD; their inclusion in model 4 therefore provides a conservative estimate and may attenuate the true association.

Subgroup analyses were performed to examine potential effect modification by key demographic and clinical characteristics. We tested for interactions by incorporating multiplicative interaction terms (NHR × stratification variable) into fully adjusted models, with statistical significance assessed using likelihood ratio tests (*P* < .05).

Dose–response relationships were evaluated using restricted cubic splines with 4 knots positioned at the 5th, 35th, 65th, and 95th percentiles of the NHR distribution.^[20]^ Nonlinearity was assessed by testing the null hypothesis that coefficients of the 2nd and 3rd spline transformations equal 0 simultaneously. Inflection points from the spline analysis reflect the behavior of the fitted model.

All analyses were conducted using R software (version 4.1.0; R Foundation for Statistical Computing). Statistical significance was set at *P* < .05 for all tests.

## 3. Results

### 3.1. Study population characteristics

Our analytical cohort comprised 1033 COPD patients, with a higher proportion of females (57.4%, n = 593) than males (42.6%, n = 440). Cardiovascular comorbidities were present in 199 participants (19.3%), while 834 participants (80.7%) had no documented CVD.

Baseline characteristics stratified by CVD status are presented in Table 1. Participants with CVD were significantly older, more likely to be male, and had a higher prevalence of diabetes and hypertension. Importantly, mean NHR was significantly higher in the CVD group (4.062 vs 3.206, *P* < .001). No significant between-group differences were observed for BMI, race/ethnicity, education, or PIR.

**Table 1:** Baseline characteristics of the NHANES 2007 to 2018 study sample, N (%).

Variable names	COPD group	COPD + CVD group	*P*
	N = 834	N = 199	
Age (yr)	61.2 ± 12.8	68.4 ± 10.2	<.001
Sex (%)			<.001
Female	516 (61.87)	77 (38.69)	
Male	318 (38.13)	122 (61.31)	
BMI (kg/m^2^)	31.84 ± 7.3	32.16 ± 6.8	.220
Race (%)			.681
Mexican American	57 (6.83)	11 (5.53)	
Non-Hispanic Black	156 (18.71)	39 (19.60)	
Non-Hispanic White	473 (56.71)	121 (60.80)	
Other Hispanic	89 (10.67)	16 (8.04)	
Other race	59 (7.07)	12 (6.03)	
Marital status (%)			.009
Divorced	169 (20.26)	30 (15.08)	
Living with partner	37 (4.44)	4 (2.01)	
Married	387 (46.40)	98 (49.25)	
Never married	75 (8.99)	16 (8.04)	
Separated	41 (4.92)	4 (2.01)	
Widowed	125 (14.99)	47 (23.62)	
Education level (%)			.193
9th–11th grade	135 (16.19)	37 (18.59)	
College graduate or above	120 (14.39)	28 (14.07)	
High school graduate	220 (26.38)	56 (28.14)	
<9th grade	105 (12.59)	33 (16.58)	
Some college or AA degree	254 (30.46)	45 (22.61)	
Smoking (%)			.078
No	270 (32.37)	51 (25.63)	
Yes	564 (67.63)	148 (74.37)	
Diabetes (%)			<.001
No	635 (76.14)	115 (57.79)	
Yes	199 (23.86)	84 (42.21)	
Hypertension (%)			<.001
No	379 (45.44)	36 (18.09)	
Yes	455 (54.56)	163 (81.91)	
PIR	1.797 ± 1.2	1.63 ± 1.1	.154
NHR	3.206 ± 1.8	4.062 ± 2.1	<.001

BMI = body mass index, COPD = chronic obstructive pulmonary disease, CVD = cardiovascular disease, HDL = high-density lipoprotein, NHANES = National Health and Nutrition Examination Survey, NHR = neutrophil-to- high-density lipoprotein cholesterol ratio, PIR = poverty income ratio.

### 3.2. NHR–CVD associations

Table 2 presents the associations between NHR and CVD across progressive adjustment models. When analyzed as a continuous variable, NHR demonstrated consistent positive associations across all models. In the fully adjusted model 4, each unit increase in NHR was associated with higher CVD odds ratio (OR: 1.24, 95% confidence interval [CI]: 1.11–1.38).

**Table 2:** Associations between NHR and CVD in COPD patients.

Variable	Model 1	Model 2	Model 3	Model 4
	OR (95% CI)	OR (95% CI)	OR (95% CI)	OR (95% CI)
NHR (continuous variable)	1.2421 (1.1395–1.3545)*	1.2779 (1.1610–1.4077)*	1.2695 (1.1482–1.4046)*	1.2361 (1.1119–1.3753)*
NHR (classified variable)				
*Q*1 (0.492–2.32,n = 259)	Reference	Reference	Reference	Reference
*Q*2 (2.321–3.361, n = 258)	1.4301 (0.8700–2.3731)	1.3238 (0.7840–2.2543)	1.2597 (0.7391–2.1648)	1.1620 (0.6741–2.0176)
*Q*3 (3.362–4.677, n = 258)	1.9015 (1.1816–3.1054)*	1.9509 (1.1667–3.3072)*	1.8310 (1.0814–3.1397)*	1.6463 (1.0357–2.8530)*
*Q*4 (4.679–8.624, n = 258)	2.9002 (1.8429–4.6624)*	3.1506 (1.9189–5.2722)*	2.9812 (1.7820–5.0782)*	2.6041 (1.5239–4.5249)*
*P* for trend	<.001	<.001	<.001	<.001

Model 1 is unadjusted. Model 2 adjusts for sex, age, and race/ethnicity. Model 3 further adjusts for education level, marital status, smoking status, and poverty income ratio. Finally, model 4 incorporates additional adjustments for hypertension, diabetes mellitus, and body mass index on top of the variables in Model 3.

CI = confidence interval, COPD = chronic obstructive pulmonary disease, CVD = cardiovascular disease, NHR = neutrophil-to- high-density lipoprotein cholesterol ratio, OR = odds ratio.

* *P* < .05.

Quartile-based analysis revealed a nonlinear pattern, with higher CVD odds emerging from *Q*3 onward. Using *Q*1 as the reference, *Q*2 showed no significant association in the fully adjusted model (OR: 1.16, 95% CI: 0.67–2.02). *Q*3 demonstrated an OR of 1.65 (95% CI: 1.04–2.85), while *Q*4 exhibited the strongest association with an OR of 2.60 (95% CI: 1.52–4.52). The test for trend across quartiles was highly significant (*P* < .001).

### 3.3. Dose–response characterization

**Figure 2.**
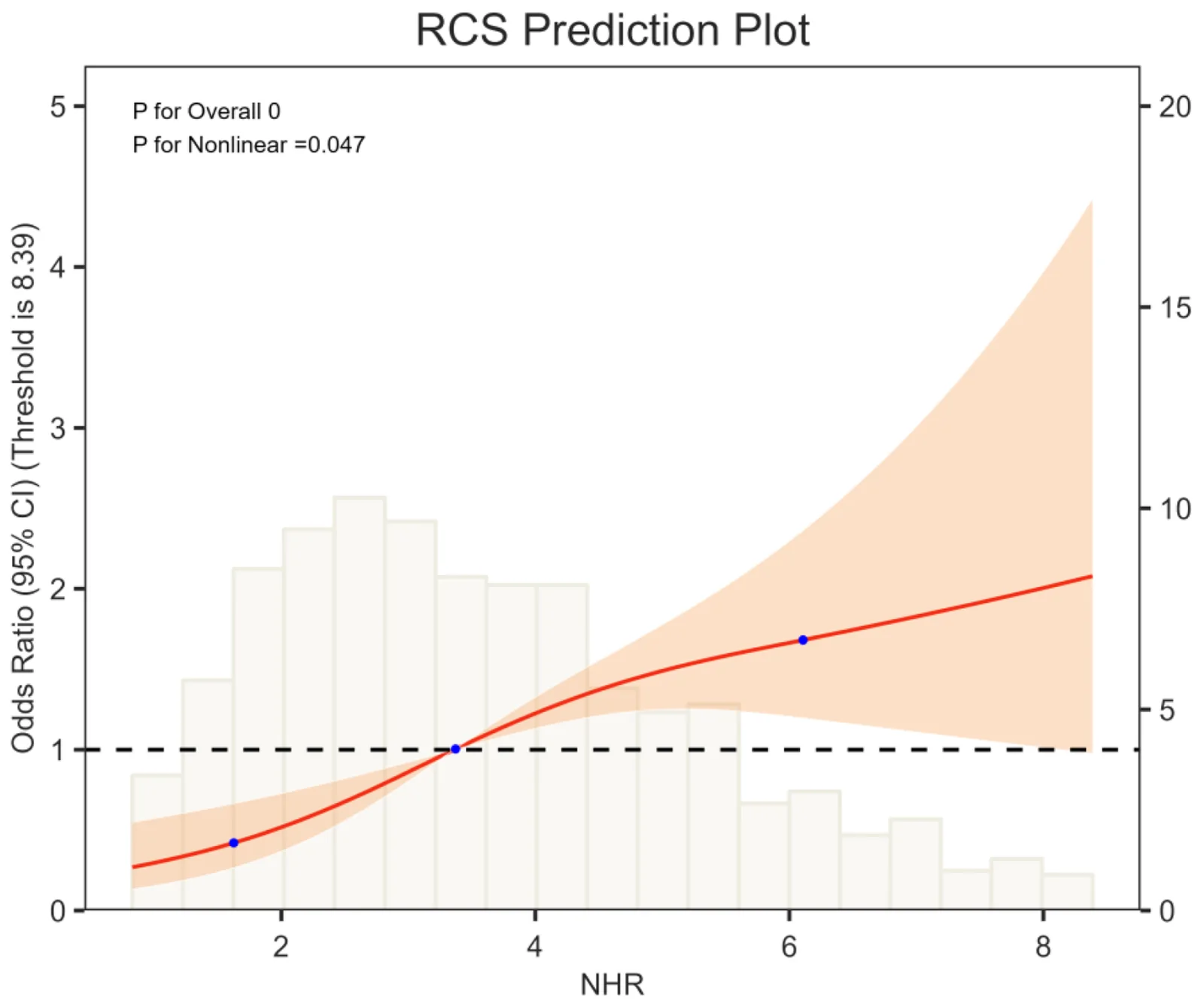
Restricted cubic spline of the association between NHR and CVD odds (fully adjusted model). CI = confidence interval, CVD = cardiovascular disease, NHR = neutrophil-to- high-density lipoprotein cholesterol ratio, RCS = restricted cubic spline.

Restricted cubic spline analysis (Fig. 2) revealed a nonlinear dose–response relationship between NHR and CVD odds. The relationship exhibited relatively modest associations at lower NHR values, followed by a steeper increase at higher NHR concentrations, consistent with the quartile analyses.

### 3.4. Subgroup analysis results

**Figure 3.**
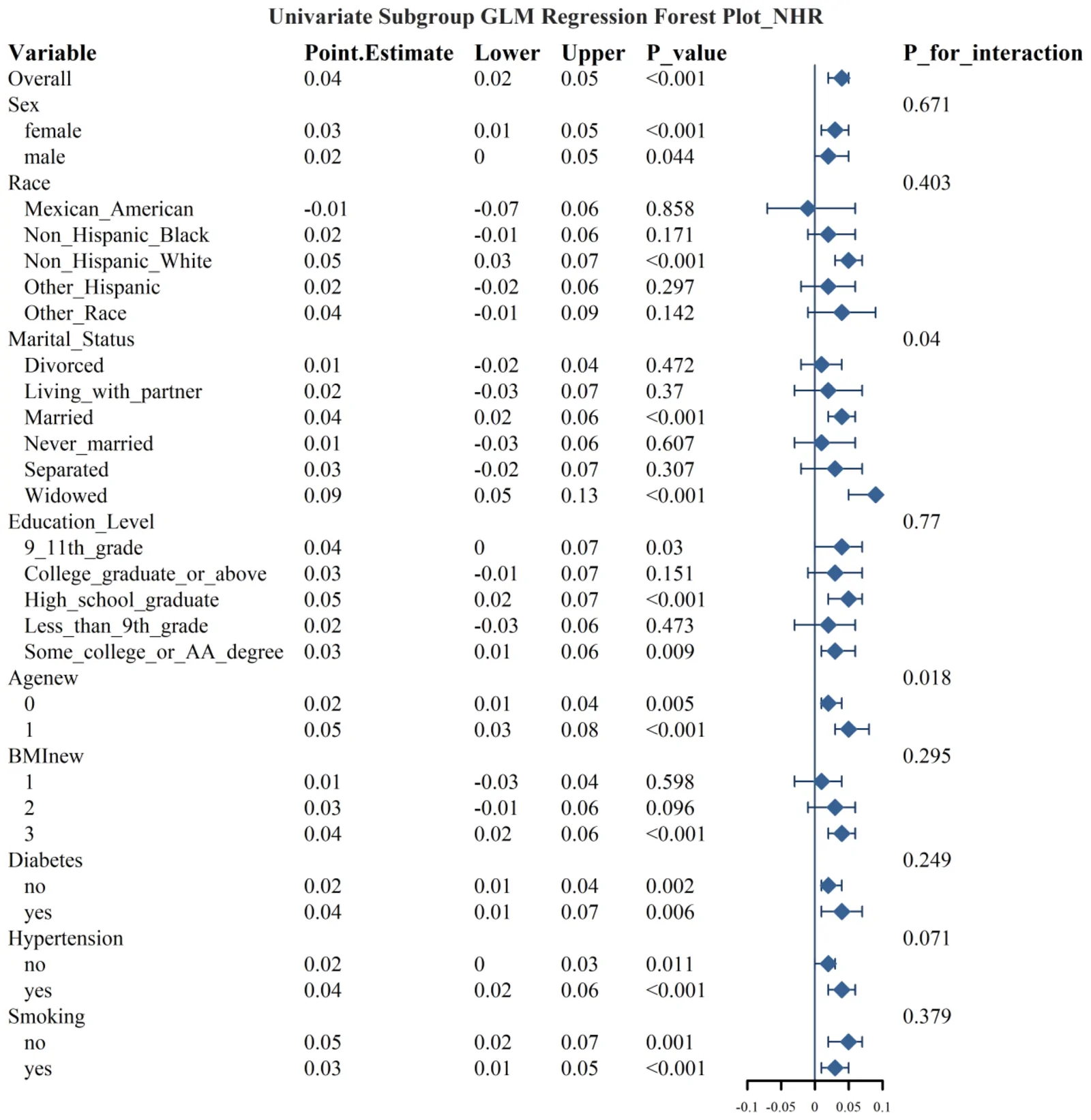
Subgroup analysis of the NHR–CVD association by demographic and clinical strata (fully adjusted model). CVD = cardiovascular disease, GLM = generalized linear model, NHR = neutrophil-to- high-density lipoprotein cholesterol ratio.

Subgroup analyses (Fig. 3) demonstrated consistent NHR–CVD associations across demographic and clinical strata. No significant interactions were detected (all *P* interaction >.05), suggesting a consistent direction of association across the population subgroups examined.

## 4. Discussion

In this cross-sectional analysis of 1033 COPD patients from NHANES 2007 to 2018, elevated NHR was independently associated with higher CVD prevalence. Participants in the highest NHR quartile had higher odds of CVD (OR: 2.60, 95% CI: 1.52–4.52) compared to the lowest quartile after multivariable adjustment. This provides the 1st evidence of an NHR–CVD association in COPD patients.

### 4.1. Principal findings

Each unit increase in NHR was associated with higher CVD odds (OR: 1.24, 95% CI: 1.11–1.38). The quartile analysis revealed a nonlinear pattern: *Q*2 (NHR: 2.32–3.36) showed no association, while *Q*3 (NHR: 3.36–4.68; OR: 1.65, 95% CI: 1.04–2.85) and *Q*4 (NHR: 4.68–8.62; OR: 2.60, 95% CI: 1.52–4.52) demonstrated progressively stronger associations.

This nonlinear pattern is consistent with observations previously reported for other inflammatory markers, in which dose–response relationships with cardiovascular outcomes are often nonlinear rather than strictly graded. The spline analysis identified a potential inflection point around NHR ≈ 3.4; however, this reflects the behavior of the fitted statistical model and should not be interpreted as a clinically validated threshold.

The association persisted after adjusting for age, sex, hypertension, diabetes, and obesity, suggesting that NHR captures information about cardiovascular comorbidity that is not fully reflected by traditional risk factors. This may be particularly relevant in COPD, where conventional cardiovascular risk equations are known to underperform due to the disease’s inflammatory profile.^[21]^

### 4.2. Mechanistic considerations

Several biological mechanisms may underlie the observed NHR–CVD association. Neutrophils drive COPD pathogenesis through the release of proteases, myeloperoxidase, and neutrophil extracellular traps.^[22,23]^ These same mechanisms also promote endothelial dysfunction and atherosclerosis. Recent reviews have summarized how neutrophil extracellular traps directly contribute to thrombus formation and atherothrombosis.^[24]^ These prothrombotic and pro-inflammatory mechanisms are directly relevant to cardiovascular comorbidity in COPD.

HDL-C exhibits antiatherogenic properties including reverse cholesterol transport, anti-inflammatory effects, and endothelial protection.^[25]^ However, chronic inflammation in COPD may impair HDL function through oxidative modification.^[26]^ Additionally, chronic corticosteroid use, common in COPD management, has been associated with dyslipidemia and impaired HDL functionality.^[27]^

NHR integrates these pro-inflammatory (neutrophil) and anti-inflammatory (HDL) pathways. In our analysis, the NHR–CVD association persisted after adjusting for traditional cardiovascular risk factors, including hypertension, diabetes, and obesity. This may suggest, although not directly tested in the present study, that NHR reflects inflammatory mechanisms not fully captured by these traditional factors – a possibility that may be particularly relevant in COPD, where systemic inflammation plays a central role in cardiovascular comorbidity.

### 4.3. Comparison with prior studies

No previous studies have examined NHR specifically in COPD populations. Our cross-sectional findings are broadly consistent with prior longitudinal research in acute coronary syndromes, heart failure, and stroke, in which elevated NHR was associated with adverse outcomes. For example, Guo et al reported that elevated NHR independently predicted target lesion restenosis and adverse cardiovascular events in patients undergoing percutaneous coronary intervention.^[28]^ Although these prior studies and ours used different designs and outcomes, the consistent direction of association supports a link between NHR and cardiovascular conditions across populations.

Prior research on related hematologic ratios also points to a role of inflammatory indices in COPD. Using longitudinal NHANES data, Ye et al reported that elevated neutrophil-to-lymphocyte ratio independently predicted cardiovascular mortality in COPD patients across demographic subgroups.^[29]^ While our cross-sectional findings cannot speak to mortality outcomes, the consistent direction of association supports a broader contribution of neutrophil-mediated inflammation to cardiovascular comorbidity in COPD. Whether incorporating HDL-C into the ratio offers additional mechanistic specificity remains to be tested in head-to-head comparative studies.

### 4.4. Subgroup findings

Exploratory subgroup analyses showed consistent directions of association across demographic and clinical strata, with no statistically significant interactions. While these findings suggest the association may be present across diverse patient subgroups, the subgroup analyses were not prespecified and should be interpreted cautiously.

### 4.5. Clinical implications

NHR is calculated from routine laboratory parameters, making it potentially accessible and cost-effective. However, clinical implementation cannot be recommended based on cross-sectional data alone. Prospective studies are needed to determine whether NHR predicts incident cardiovascular events in COPD patients and whether NHR-guided interventions improve outcomes. Such validation is essential before NHR can be considered for clinical use.

### 4.6. Limitations

Several limitations warrant consideration. First, the cross-sectional design precludes causal inference and does not allow assessment of temporal relationships between NHR and CVD development. Second, both COPD and CVD were identified through self-reported physician diagnoses rather than objective measures such as spirometry or cardiac imaging, which may result in misclassification. While this approach is standard in NHANES analyses and demonstrates acceptable concordance with objective measures in population studies, underdiagnosis or recall bias cannot be excluded, and such nondifferential misclassification would most likely bias the observed association toward the null. Third, the inflection point identified through spline analysis (NHR ≈ 3.4) reflects the behavior of the fitted statistical model rather than a clinically validated threshold and requires prospective validation before clinical application. Fourth, data on COPD severity (global initiative for chronic obstructive lung disease stage), specific medications, and CVD subtypes were unavailable, limiting our ability to assess disease-specific associations. Fifth, functional HDL measures (e.g., cholesterol efflux capacity) would be more informative than HDL concentration alone in capturing anti-inflammatory properties. Finally, residual confounding by unmeasured factors (diet, physical activity, and genetics) cannot be excluded despite multivariable adjustment.

## 5. Conclusions

In this cross-sectional NHANES analysis, elevated NHR was independently associated with a higher cardiovascular disease prevalence in COPD patients. The association remained consistent across different modeling approaches and demographic subgroups. Spline analysis revealed a nonlinear pattern with a potential inflection point around NHR ≈ 3.4; however, this reflects the behavior of the fitted statistical model rather than a clinically validated threshold. As NHR is derived from routine laboratory measurements, prospective studies are warranted to determine whether it predicts incident cardiovascular events and could inform cardiovascular evaluation in COPD populations.

## Acknowledgments

The authors thank the National Center for Health Statistics and the Centers for Disease Control and Prevention for designing and conducting NHANES and making the data publicly available. We also appreciate the participants of NHANES for their valuable contributions to this research.

## Author contributions

**Conceptualization:** Xia Zhang.

**Data curation:** Xia Zhang.

**Investigation:** Juhua Duan.

**Writing – original draft:** Xia Zhang.

**Writing – review & editing:** Juhua Duan.
